# Effects of Two Different *Rhodiola rosea* Extracts on Primary Human Visceral Adipocytes

**DOI:** 10.3390/molecules20058409

**Published:** 2015-05-11

**Authors:** Elena Pomari, Bruno Stefanon, Monica Colitti

**Affiliations:** Department of Agricultural and Environmental Sciences, University of Udine, via delle Scienze, 206, 33100 Udine, Italy; E-Mails: elena.pomari@gmail.com (E.P.); bruno.stefanon@uniud.it (B.S.)

**Keywords:** human visceral adipocytes, differentiation, gene expression, *Rhodiola rosea*

## Abstract

*Rhodiola rosea* (*Rro*) has been reported to have various pharmacological properties, including anti-fatigue, anti-stress and anti-inflammatory activity. It is also known to improve glucose and lipid metabolism, but the effects of *Rhodiola rosea* on adipocyte differentiation and metabolism are not still elucidated. In this study the anti-adipogenic and lipolytic activity of two extracts of *Rhodiola rosea*, containing 3% salidroside (RS) or 1% salidroside and 3% rosavines (RR) on primary human visceral adipocytes was investigated. Pre-adipocytes were analyzed after 10 and 20 days of treatment during differentiation and after 7 days of treatment when they reached mature shape. The RS extract significantly induced higher apoptosis and lipolysis in comparison to control cells and to RR extract. In contrast, RR extract significantly reduced triglyceride incorporation during maturation. Differentiation of pre-adipocytes in the presence of RS and RR extracts showed a significant decrease in expression of genes involved in adipocyte function such as SLC2A4 and the adipogenic factor FGF2 and significant increase in expression of genes involved in inhibition of adipogenesis, such as GATA3, WNT3A, WNT10B. Furthermore RR extract, in contrast to RS, significantly down-regulates PPARG, the master regulator of adipogenesis and FABP4. These data support the lipolytic and anti-adipogenetic activity of two different commercial extracts of *Rhodiola rosea* in primary human visceral pre-adipocytes during differentiation.

## 1. Introduction

Many natural phytonutrients have been recognized to have beneficial effects on health and several botanicals have received positive attention for their antiadipogenic and metabolic effects in animals and humans [1,2]. The increase of body fat is due to an imbalance between energy intake and energy expenditure and natural products can be useful to decrease lipid absorption, energy intake, lipogenesis, pre-adipocyte differentiation and proliferation, or to increase energy expenditure and lipolysis [3,4]. These properties are reported for specific flavonoids [5], such as chlorogenic acid from green coffee bean [6] and carnosic acid in rosemary [7,8]. Among the plants with favorable bioactivity on fat tissue and metabolism, *Rhodiola rosea* (*Rro*), a popular plant in the Nordic countries, Eastern Europe and Asia, has gained attention in the past and its liquid extract has been produced industrially in Russia since 1975 [9]. More recently, *Rro* was registered in the UK as a traditional herbal medicinal product for use as anadaptogen [10]. The *Rro* belongs to the family of Crassulaceae, genus *Rhodiola*, and includes more than 100 different species, at least 20 of which are used in traditional Asian medicine [11,12]. However, animal and human studies have been conducted on *Rro*, so whether other species confer the same health benefits is unknown [13,14].

Phytochemical studies on the *Rro* root have shown the presence of six groups of compounds: phenylpropanoids (rosin, rosavin, rosarin), phenylethanol derivatives (salidroside, tyrosol), flavonoids (acetylrodalgin, rodiolin, rodionin, rodiosin, tricin), monoterpernes (rosaridin, rosiridol), triterpenes (β-sitosterol, daucosterol) and phenolic acids (chlorogenic hydroxycinnamic and gallic acids) [15,16]. According to the Soviet Pharmacopeia [17], the extracts of *Rro* are standardized for both rosavins and salidroside (*p*-hydroxyphenylethyl-*O*-β-d-glucopyranoside). *Rro* extracts used in human clinical studies are often standardized to minimum 3% rosavins and 0.8%–1% salidroside, because the ratio of these compounds in *Rro* root is approximately 3:1 [14]. Although Brown *et al.* [11] considered that standardized *Rro* extract should contain the full spectrum of pharmacologically active compounds, including not only salidroside and *p*-tyrosol, but also rosavin, rosin, and rosarin, many suppliers have only standardized their products to 1% salidroside. Meanwhile, according to Chang *et al.* [18], salidroside is considered to be one of the major phenolic glycosides in *Rro*, and is generally used to evaluate the quality of *Rhodiola*.

The main effect described of genus *Rhodiola* is adaptogenic, meaning that in normal doses the products are non-toxic, produce a non-specific response and have a normalizing physiologic influence and stress protective activity [14]. *Rro* is mainly known for stimulating physical endurance, attention span, memory, and work productivity [19,20,21], whereas its bioactivity on adipocytes is poorly characterized. Apart the known antioxidant [22,23,24], antitumour [25,26], antidepressive [27,28], neuroprotective [29,30], cardioprotective [31,32] hepatoprotective [33,34], and immunostimulating effects [35,36,37], *Rro* has been recently described for its ability to regulate blood sugar levels in diabetics and to activate the lipolytic processes [38,39]. Moreover, *Rro* standardized for 3% rosavins and 1% salidroside, in combination with *Citrus aurantium*,has been indicated to mobilize lipids from adipose tissue for weight reduction [40]. *Rro* plus *Citrus aurantium* has been found to decrease visceral fat weight by 30% of rats fed with high fat diet, having a direct effect on sympathetic tone and on hypothalamic norepinephrine secretion [40]. Ethanol soluble fraction of *Rro*, not clearly characterized for its content, has been demonstrated to induce peroxisome proliferator-activated receptor delta (PPARδ) expression in cardiomyocytes [32]. PPARδ is a homologue of PPARγ, and plays an important role in many tissues such as brain, skin, muscles and adipocytes [41]. Peroxisome proliferator-activated receptor beta (PPARβ) and PPARδ prevent triglyceride accumulation and increases lipid catabolism in adipocytes [42]. In addition, PPARδ increases thermogenesis by up-regulation of the expression of hormone-sensitive lipase (HSL) and uncoupling protein 1 (UCP1) [42,43]. If the action of *Rro* on PPARβ/δ expression in adipocytes corresponded to that in cardiomyocytes, a reduction of lipogenesis and an increase of lipolysis could be expected also in adipose tissue cells.

In a recent paper, El-Houri *et al.* [44] have reported that only the dichloromethane extract of aerial parts of *Rro*, but not methanol extract, triggered PPARG transactivation without stimulating adipocyte differentiation. Conversely, either dichloromethane or methanol root extracts inhibited fat accumulation in the *C. elegans* model. The type of solvent and the extraction procedures applied to root or aerial parts produce extracts of variable composition and consequently with diverse bioactivities [44].

Clinical trials performed in the Russian Federation have provided interesting evidence such as the fact oral administration of 200 mg *Rro* extract with rosavin activates HSL and mobilizes fatty acids from adipose tissue in healthy volunteers and obese patients [45,46]. Moreover, it has been demonstrated that rosiridin, a bio-active compound isolated from *Rro*, interferes with the degradation of norepinephrine that regulates the HSL activity by inhibiting the action of monoamine oxidases (MAOs) [47] and breaks down fat stored in adipose tissue.

Evaluating the present knowledge, this interesting adaptogenic plant could be useful to reduce or prevent adipogenesis and to support weight loss. However *in vitro* scientific research at the cellular and molecular levels are required to explain and to confirm the benefits of *Rro* on lipid metabolism and adipogenesis. Considering the different spectra of extracts contained in *Rhodiola* preparations, the present research aims to identify possible effects on primary human omental pre-adipocytes and mature adipocytes. Moreover, two different extracts, one (RS) standardized for salidroside (≥3%) and the other (RR) standardized for salidroside (≥1%) and rosavines (≥3%) were compared. In particular, the effects of RS and RR extracts were tested *in vitro* on pre-adipocyte and adipocyte viability, apoptosis, lipolysis and adipogenesis. In addition, expression levels of genes involved in the human adipogenesis pathway during pre-adipocyte differentiation were analyzed by PCR array.

## 2. Results and Discussion

### 2.1. Cell Viability

A viability assay was used to find out the highest dose of RR and RS extracts that allowed a cell viability over 60%. After treating P10, P20 and A7 cells with 5, 10, 30 or 70 µg/mL RR and RS extracts, the data showed that cell viability on P10 and P20 cells significantly (*p* < 0.001) decreased in a dose-dependent manner (Table 1), and that the viability measured at a dose of 70 µg/mL was always significantly different from other doses. Nevertheless, viability of all cells except A7 remained over 60% up to 30 µg/mL dose and markedly decreased at 70 µg/mL. In A7 cells viability remained over 80% up to 70 µg/mL dose. According to these evidences, 30 µg/mL of plant extract was chosen for the experiments.

**Table 1:** Modulation of MTT metabolism by RS or RR extracts in human omental pre-adipocytes. Cells were treated with different doses of extracts. P10 differentiating pre-adipocytes treated for 10 days; P20 differentiating pre-adipocytes treated for 20 days; A7, mature adipocytes treated for 7 days. Data are expressed as percentage of control cells (untreated) and presented as means ± standard deviation (SD). Different superscript capital letters indicate significant differences (*p* < 0.001) within treatments at different concentrations.

Dose (µg/mL)	P10		P20		A7	
	RS	RR	RS	RR	RS	RR
5	91.83 ^A^ ± 7.55	91.51 ^A^ ± 7.58	91.34 ^A^ ± 3.26	89.41 ^A^ ± 3.42	96.32 ^A^ ± 1.71	96.74 ^A^ ± 1.83
10	80.26 ^B^ ± 6.84	80.17 ^B^ ± 7.01	78.52 ^B^ ± 2.43	79.02 ^B^ ± 3.13	95.81 ^A^ ± 2.05	96.29 ^A^ ± 1.92
30	76.75 ^C^ ± 6.43	79.95 ^B^ ± 6.78	66.85 ^C^ ± 2.88	67.77 ^C^ ± 3.33	96.09 ^A^ ± 2.25	93.84 ^A^ ± 2.25
70	53.58 ^D^ ± 7.15	55.30 ^C^ ± 4.23	36.26 ^D^ ± 2.06	42.28 ^D^ ± 4.33	89.81 ^B^ ± 1.91	84.34 ^B^ ± 3.06

#### 2.1.1. RR and RS Extracts Decrease Triglyceride Accumulation

**Figure 1:**
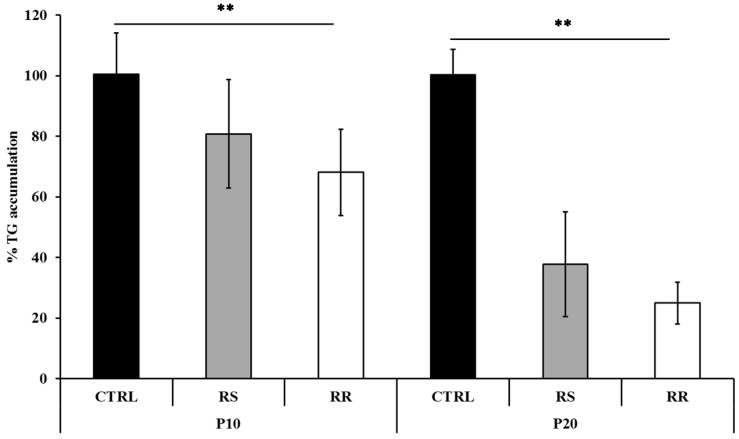
Effects of RS and RR extracts on triglyceride accumulation during pre-adipocyte differentiation. Triglyceride accumulation of differentiating pre-adipocytes incubated for 10 d (P10) and 20 d (P20) with RS and RR extracts relative to untreated control cells (CTRL) set as 100%. Results are depicted as mean ± standard deviation (SD). Asterisks ** indicate the significant difference between treatments for *p* < 0.001.

The ability of RS and RR extracts to prevent triglyceride accumulation was demonstrated on P10 and P20 cells treated with 30 µg/mL extracts and on the corresponding CTRL cells. The total amount of lipid accumulation was reported as a percentage respect to CTRL (where CTRL was considered as 100%, Figure 1). 

The data indicate that the tested compounds, compared to CTRL, inhibit adipogenesis at P10 and P20, exhibiting a significant (*p* < 0.001) decrease of triglyceride levels compared to CTRL. Triglyceride accumulation was significantly (*p* < 0.001) lower in cells treated with RR (68.08 ± 14.27 at P10 and 24.99 ± 6.91 at P20), in comparison to cells treated with RS (80.77 ± 17.91 at P10 and 37.80 ± 17.27 at P20). The interaction between cells at different stages and treatments was also significant (*p* < 0.001).

#### 2.1.2. RR and RS Extracts Increase Glycerol Release

**Figure 2:**
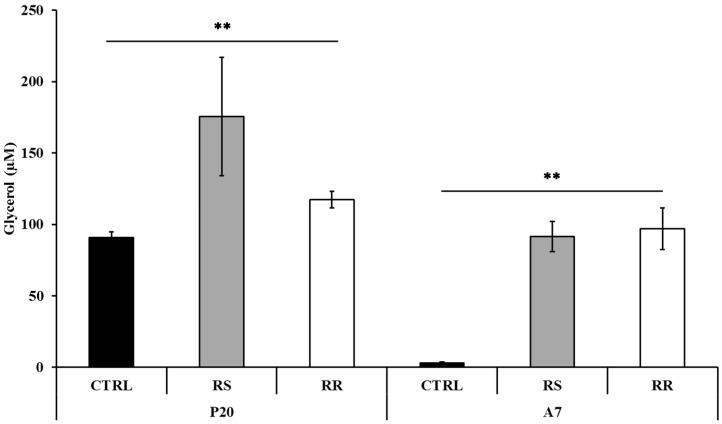
Determination of glycerol release in differentiating (P20) and mature (A7) adipocytes after incubation with RS and RR extracts where glycerol content is given in μM. Results are depicted as mean ± standard deviation (SD). Asterisks ** indicate the significant difference between treatments for *p* < 0.001.

The lipolysis activity was assessed on P20 and A7 cells treated with 30 µg/mL RR and RS extracts and on the corresponding CTRL cells. Treatment of P20 cells with RS extract significantly (*p* < 0.001) incremented the content of free glycerol in the culture medium to 175.47 μM (±41.3) as compared to 117.31 μM (±5.6) in RR-treated cells and 90.7 μM (±3.9) in CTRL cells (Figure 2). On the contrary, the treatment of A7 cells with RR extract significantly (*p* < 0.001) increased the release of free glycerol to 96.8 μM (±14.7) in comparison to RS-treated cells (91.5 μM (±10.5)) and to CTRL cells (2.9 μM (±0.7)) (Figure 2). The interaction between cells at different stages and treatments was also significant (*p* < 0.001).

#### 2.1.3. Effect of RR and RS on Apoptosis

**Figure 3:**
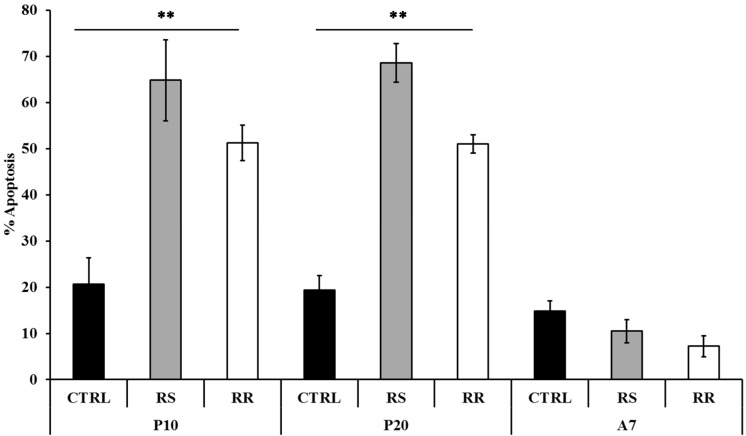
Modulation of apoptosis by RS and RR extracts in human omental pre-adipocytes. Cells were treated with 30 µg/mL RR and RS extracts. P10, differentiating pre-adipoctes treated for 10 d; P20, differentiating pre-adipocytes treated for 20 d; A7, mature adipocytes treated for 7 d. Data are presented as mean ± standard deviation (SD). Asterisks ** indicate the significant difference between treatments for *p* < 0.001 as percentage vs positive CTRL.

The apoptotic effect of the extracts was examined on P10, P20 and A7 cells treated with 30 µg/mL RS and RR and on corresponding CTRL cells. A significant (*p* < 0.001) increase of the percentage of apoptosis in P10 and P20 cells under treatment with RR and RS extracts was observed (Figure 3). On the contrary, the apoptotic percentage on A7 cells did not significantly vary between treatments. The interaction between cells at different stages and treatments was also significant (*p* < 0.001). Interestingly, the percentage of apoptosis induced by RS extract (64.84% ± 8.81% at P10 and 68.61% ± 4.17% at P20) was significantly (*p* < 0.001) higher than that induced by RR extract (51.25% ± 3.85% at P10 and 59.98% ± 1.98% at P20).

**Figure 4:**
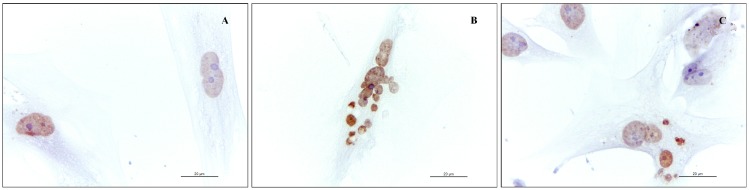
Cytological visualization of apoptosis by TUNEL assay on P20 pre-adipocytes. Nuclei of apoptotic cells are marked in brown, counterstained with Gill’s hematoxylin. (**A**) CTRL cells; the nuclei are clearly positive, partly demonstrating alteration of nuclear membrane; (**B**) RS-treated pre-adipocytes; extrusion of apoptotic nucleus, presence of apoptotic bodies and nucleus fragmentation; (**C**) RR-treated pre-adipocytes; positive nuclei with condensed chromatin and apoptotic remnants.

Morphological characteristics of P20 cells, labelled by TUNEL assay, are shown in Figure 4. Nuclei of apoptotic cells were marked in brown colour and showed chromatin condensation with a diffuse increase in nuclear density and parallel loss of nuclear volume (Figure 4A,C). These features were followed by fragmentation of cell and its nucleus, resulting in smaller apoptotic bodies (Figure 4B).

#### 2.1.4. Effects of RR and RS Extracts on the Level of Expression of Adipogenesis-Associated Genes

**Figure 5:**
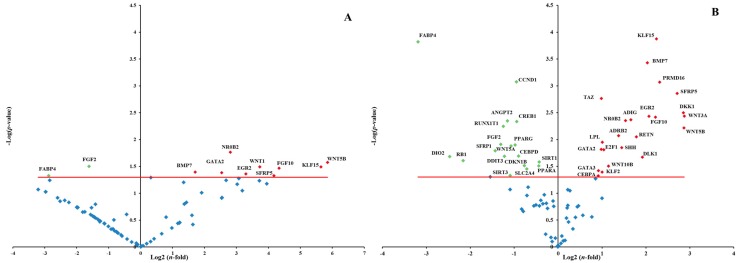
Volcano plot of adipogenesis PCR array. PCR array analysis of gene expression in RS and RR-treated P20 cells in comparison to P20 CTRL cells. (**A**) Gene expression of P20 RS-treated cells. (**B**) Gene expression of P20 RR-treated cells. Total RNA from three independent experiments, one per each donor, was isolated from both CTRL cells and cells treated with RR or RS extracts incubation. Cells were used at the third passage. The relative expression levels for each gene depicted as log_2_(*n*-fold) are plotted against −Log(*p*-value). Red indicator = significantly up-regulated gene; Green indicator = significantly down-regulated gene. Red line indicates −Log(*p*-value), *p* < 0.05.

The expression pattern of genes involved in the adipogenesis pathways was measured on P20 treated cells using a human RT^2^ Profiler PCR Array. Volcano plot reported the log_2_(*n*-fold) values of significantly (*p* < 0.05) up- and down-regulated genes in comparison to CTRL cells (Figure 5). The results revealed that RS extract modulates the expression of 13% (11/84) of the genes (Figure 5A) and RR extract modulates the expression of 50% (42/84) of the genes (Figure 5B).

Among the genes involved in the adipogenesis, CAMP responsive element binding protein 1 (CREB1), peroxisome proliferator-activated receptor, gamma 2 (PPARG), fibroblast growth factor 2 (FGF2), retinoblastoma 1 (RB1), CCAAT/enhancer binding protein, delta (CEBPD), cyclin D1 (CCND1), solute carrier family 2, member 4 (SLC2A4), sirtuin 3 (SIRT3), secreted frizzled-related protein 1 (SFRP1) and fatty acid binding protein 4 (FABP4) were significantly (*p* < 0.05) down-regulated from RR extract (30 μg/mL). Among the genes involved in inhibition of adipogenesis, delta-like 1 homolog (DLK1), GATA binding protein 2 (GATA2), GATA binding protein 3 (GATA3), Kruppel-like factor 2 (KLF2), wingless-related MMTV integration site 10B (WNT10B), wingless-related MMTV integration site 3A (WNT3A), sonic hedgehog (SHH), adrenoceptor beta 2 (ADRB2) and tafazzin (TAZ) were significantly (*p* < 0.05) up-regulated.

Treatment with RS extract (30 µg/mL) up-regulated (*p* < 0.05) the expression of wingless-type MMTV integration site family, member 1 (WNT1) and of the genes which were similarly modulated by RR extract (WNT5B, KLF15, FGF10, SFRP5, EGR2, NR0B2, BMP7), one of which, GATA2 (GATA binding protein 2), involved in inhibition of adipogenesis. The expression of FGF2 and FABP4 involved in inhibiting adipogenesis, significantly (*p* < 0.05) decreased also in RS-treated cells.

### 2.2. Discussion

Adipogenesis is a cell differentiation regulated by a profusion of transcription factors and cell-cycle proteins that regulate gene expression and lead to mature adipocytes. In this network regulators that activate or inhibit the transformation of cells from fibroblastic to spherical shape are involved [48]. The obesity is thus related to an increase of number and size of adipocytes that takes place in association with positive energy balance.

Research in nutrition to find natural products that, targeting adipocytes and their pathways, could lead to induction of lipolysis and apoptosis or to inhibition of adipogenesis has been recently aroused [4,8]. Given the numerous effects of *Rro*, including the influence on feeding behavior [49], and the different available content of bioactive compounds, one of the objectives of the present study was to compare the effects of two different extracts of *Rro* in preventing adipogenesis and in modifying adipose cells metabolism on human primary visceral adipocytes.

The ability of RS and RR extract to prevent lipid accumulation was examined with Oil Red O staining and triglyceride content on P10 and P20 human primary omental pre-adipocytes. The reduction of triglyceride incorporation during differentiation of RS and RR treated cells (Figure 1) was coincident with an enhancement of lipolytic activity, as detected by an increase of glycerol release, in P20 RS-treated cells and in A7 RR-treated adipocytes (Figure 2). Lipolytic activity has been reported to rosavines, cynnamic glycosides [50], that are demonstrated to stimulate lipoprotein lipase (LPL). However, it should be noted that RS extract was significantly (*p* < 0.001) more effective than RR extract in inducing apoptosis both on P10 and P20 cells, whereas the treatment of A7 adipocytes did not lead to any difference in comparison to CRTL cells (Figure 3). The lack of response of mature adipocytes (A7) to cell viability treatments (Table 1) corresponds to previous observations on mature human adipocytes exposed to *Rosmarinus officinalis* extract [8]. Studies on animal, conducted with *R. crenulata* standardized for 1.1% salidroside, *p*-tyrosol (0.3183%), *trans*-caffeic acid (0.036%) and kenposide A (0.0195%), have been reported to improve glucose and lipid metabolic disturbance in Zucker diabetic fatty (ZDF) rats [51]. Although a detailed molecular mechanism of the lipid lowering and anti-inflammation effects of salidroside alone has not yet been identified, *in vivo* studies on high fat diet-fed LDLr^−/−^ mice demonstrated that this compound reduced serum lipid levels and decreased atherosclerotic plaque formation [52]. The hypolipidemic activity of *Rro* in Winstar rats fed with hypercholesterolemic diet was also reported [53]. In fact, RS extract significantly affected lipolysis and apoptosis in comparison to RR extract that was more effective in reducing differentiation. This distinctive bioactivity of two extracts can be due to the lower concentration of salidroside in RR or due to the different composition of its phytocomplex that contains also rosavines and therefore deserves further studies. A different modulation of gene expression in adipogenesis-related genes between extracts was also found during differentiation of pre-adipocytes (P20) (Figure 5 and SM Table 1).

To date, only one paper reported the effect of salidroside on the differentiation of 3T3-L1 adipocytes, using in this biological research the mouse embryonic fibroblast cell line [54]. In this cell line, salidroside promoted the 3H-glucose uptake, significantly suppressed the differentiation, down-regulated the expression of peroxisome proliferator-activated receptor gamma (PPARγ) and CCAAT-enhancer-binding proteins alpha (C/EBPα) mRNA [54]. Interestingly, salidroside has been proven to have curative proprieties in bingeing-related eating disorders in rat models [55]. Since binge eating was evoked by combining stress and repeated episodes of food restriction, the effects of salidroside on this experimental model could be considered as an indirect approach to treat the energy intake.

Genes that inhibit the pre-adipocyte to adipocyte transition, including members of the GATA-binding proteins and WNT family of secreted glycoproteins, were significantly (*p* < 0.05) up-regulated in P20 treated cells. It is known that continuous expression of the binding proteins GATA2 and GATA3 can inhibit the differentiation in pre-adipocytes [56]. The enhanced expression of GATA2 and GATA3 suppresses adipocyte differentiation through the reduced PPARG and CEBPs activity [57]. The *in vitro* data revealed that both extracts up-regulate the GATA2 expression and RR extract affects also the expression of GATA3 (Figure 5). It has been observed that berberine and evodiamine, two botanical alkaloids [58], and also rosemary extract [8] up-regulate the expression of GATAs and influence the PPARG and CEBPA expression. Therefore, the expression of GATAs may contribute to the observed down-regulation of PPARG. Moreover, in P20 RR-treated cells the repression of PPARG may be mediated by the over-expression of KLF2, has been also recognized as anti-adipogenetic factor, repressing PPARG promoter [59]. This was also observed for *yerba maté* and resveratrol on 3T3-L1 cell line [60]. However, in both P20 RR- and RS-treated cells, KLF15, which promotes adipocytes differentiation and is induced in the early stages of adipocyte differentiation [61] was significantly up-regulated. In the present study, KLF15 was not activated by CEBPB and CEBPD, which was significantly down-regulated in RR-treated cells (Figure 5 and SM Table1). The results obtained for RR-treated cells are in the agreement with the loss of SLC2A4 expression, that is usually a target of KLF15 [62].

In addition, it is known that the CEBPs and PPARG expression depends on DLK1, also known as preadipocyte factor 1 (Pref-1), and it is widely accepted that DLK1 plays an important role in adipocytes differentiation [63]. Gene expression analysis of P20 RR-treated cells showed over-expression of DLK1 as well as up-regulation of the transcriptional co-activator with PDZ-binding motif (TAZ), known as co-repressor of PPARG in adipose tissue. The observed down-regulation of PPARG and CEBPD led to a reduced expression of key genes involved in downstream differentiation (FABP4, SLC2A4), and induced phenotypic changes as triglyceride accumulation.

Wnts are a broad family of proteins that participate in several cellular biological processes, and it is reported that Wnt signaling has a role in preventing adipocyte differentiation [64]. WNT6, WNT10A, and WNT10B are expressed in precursor cells and decline during differentiation blocking adipogenic conversion of 3T3-L1 pre-adipocytes through stabilization of β-catenin and inhibition of CEBPA and PPARG [65]. In contrast, WNT5B and WNT4 are transiently induced during adipogenesis and act to promote this process [66]. A further research suggests that, in order to elicit its antiadipogenic effects, the canonical ligand WNT3A among several others inhibits activation of both PPARG and CEBPA [67]. As recently observed, WNT3A, an inhibitor of pre-adipocyte to adipocyte transition, was significantly up-regulated in human pre-adipocytes treated with *Rosmarinus officinalis* extract [8] and in 3T3-L1 cells treated with *herba maté* [68]. In our study a significant increase in the expression of WNT3A, WNT10B, WNT5B was observed in P20 RR-treated cells in comparison to CTRL cells. In P20 RS-treated cells WNT1, WNT10B, WNT5B were significantly up-regulated as well. In particular, WNT1 was up-regulated only by RS extract and WNT10B, WNT5B were respectively up-regulated 3- and 2-fold in comparison to RR treated cells. These results may be of interest since it has been demonstrated that WNT10B mutations are associated with obesity [69] and that it blocks adipogenesis in 3T3-L1 preadipocytes *in vitro* via stabilization of free cystolic β-catenin [70]. Moreover, WNT1 has been recently recognized as an adipokine and a possible novel therapeutic target for obesity linking obesity to inflammation and insulin resistance [71].

WNT5B is known to promote adipogenesis in 3T3-L1 preadipocytes, inhibiting canonical Wnt/β-catenin signaling pathway and stimulating PPARG and FABP4 [72,73]. In P20 treated cells, the up-regulation of WNT5B did not induce PPARG nor FABP4 expression, but it is likely that the decreased expression of CCND1, which is a downstream target gene of β-catenin [74], can be related to impaired canonical Wnt pathway. Indeed even SFRPB5 and DKK1, known as Wnt pathways inhibitors, were up-regulated by RR treatment and only SFRPB5 by RS treatment. Meanwhile, SFRPB1 was significantly down-regulated by RR extract. Interestingly, the relationship between WNT5A and SFRPB5 shows some intriguing controversies. The role of SFRP5 remains unclear because it has been shown that SFRP5 was up-regulated [61] and down-regulated [75] in WAT of different obese mouse models. Furthermore, SFRP5^−/−^ mice are reported to be either resistant [61] or sensitive [75] to diet induced obesity. In addition, in human visceral adipocytes it has been demonstrated that SFRP5, binding and isolating WNT5A, prevents activation of frizzled receptors thus attenuating the noncanonical Wnt signaling [76]. In cells treated with RS extract, we observed an up-regulation of SFRP5 and a down-regulation of WNT5A and it can be speculated that the balance between WNT5A and SFRP5 expressions may act as a rheostat to control the degree of noncanonical Wnt signaling.

Transcription of genes involved in re-entry to the cell cycle of pre-adipocytes is known to be under regulation of a cascade of cell-cycle proteins such as Cdk-cyclin-E2F-Rb signaling family members [77,78]. The retinoblastoma proteins (pRb) regulate the activity of E2F transcription factors and E2F1 is strongly upregulated during the first phases of adipogenesis, when it regulates adipocyte differentiation, modulating the expression of genes such as PPARG and CCND1. In the present study, RR extract entailed an up-regulation of E2F1, but a down-regulation of upstream (RB1) and downstream targets (PPARG, CCND1). In fact, it is know that pRb can also act negatively during adipogenesis by forming a complex with PPARG [79]. Moreover, it is known that D-type cyclins represent a link between cell cycle progression, cell differentiation, and transcriptional regulation, being CCND1 repressor of PPARG expression and CCND3 activator of master regulator [80].

## 3. Experimental Section

### 3.1. Materials

Two extracts of *Rhodiola rosea* root of Chinese origin, were provided by the company nVH Italia (Cadorago, Italy) and according to the technical sheet one contained only salidroside (≥3%) (named here RS) and the other contained salidroside (≥1%) and cinnamylglycosides (rosavines) (≥3%) (named here RR). To obtain RS and RR extracts, *Rhodiola* root was dissolved in 70/30 (V/V) and 60/40 (V/V) water/ethanol solution, respectively, at 105 °C for 3 h, then the extract solution was centrifuged and the upper liquid was lyophilized, whereby the loss on drying was ≤5%. The drug extract ratio was 10:1 for RS and 6/8:1 for RR.

In the RR phytocomplex, but not in the RS, rosarin, rodosin, rosin, gallic acid, caffeic acid and chlorogenic acid were also present in unspecified amounts.

The dried extracts were stored in refrigerator at 2–8 °C until use. For further analyses then each extract (50 mg) was dissolved in 1 mL of water solution of 10% dimethylsulfoxide (DMSO), filtered with 0.22 µm pore size (Millipore, Milan, Italy) and kept in the dark at −20 °C.

### 3.2. Cell Culture and Cell Treatment

Cells and media were obtained from Zen-Bio (Research Triangle Park, NC, USA). Primary omental pre-adipocytes were collected from Caucasian normal (non-diabetic and non-smoker) women donors (*n* = 3). The mean donor age was 48.67 ± 9.07 year and mean BMI was 42.70 ± 6.95 kg/m^2^. Pre-adipocytes were cultured in omental pre-adipocytes medium (OM-PM). In order to differentiate pre-adipocytes into adipocytes, omental differentiation medium (OM-DM) was used. Differentiated adipocytes were cultured in omental adipocyte maintenance medium (OM-AM). All cells were maintained in humidified air with 5% CO_2_ at 37 °C [8].

Cells (passage 3) were treated with RR and RS extracts at increasing concentrations and during different stages of differentiation with the same final concentration of 0.014% DMSO in the culture medium. The control cells (CTRL) were incubated within the same conditions at final concentration of DMSO 0.014% in culture medium. Treatments were performed in three different stages of the cell life cycle: on pre-adipocytes for 10 days (P10) and 20 days (P20) in OM-DM, and on mature fully differentiated adipocytes for 7 days (A7) in OM-AM.

For the apoptosis, lipolysis and adipogenesis assays, cells were seeded in a 96 well plate at a density of 1 × 10^4^ cells/well. For PCR assay, cells were seeded in a 6 well plate at a density of 1 × 10^5^ cells/well and left to grow overnight. The analysis was performed using cells of the three different donors and each donor was assayed in triplicate.

#### 3.2.1. Cell Viability

The effect of the RS and RR extracts at different concentrations (0, 5, 10, 30 and 70 µg/mL) on cell viability was determined by a colorimetric assay in P10, P20 and A7 cells based on 3-(4,5-dimethylthiazol-2-yl)-2,5-diphenyltetrazolium bromide (MTT) [81]. For the MTT assay, cells were seeded in a 96 well plate at a density of 1 × 10^4^ cells/well then cells were washed with 1X PBS and fresh medium with 20 μL of MTT reagent (5 mg/mL) (Sigma, Milan, Italy) was added to each well, followed by 3 h incubation at 37 °C. After, the mixture was suctioned completely and 100 μL/well of DMSO was added to dissolve the formed formazan crystals. The absorbance was measured at 570 nm by a microplate reader and the surviving cell fraction was calculated. The cell viability was expressed as a percentage relative to CTRL cells considered as 100%.

#### 3.2.2. Oil Red O Staining and Measurement of Lipid Accumulation

In order to quantify lipid accumulation, Oil-Red O staining (ORO, Sigma) was used. The assay was performed on treated and CTRL P10 and P20 cells. After treatment with RR and RS extract (30 μg/mL) on 96 well culture plate, cells were first rinsed with PBS and then fixed with 4% formaldehyde for 30 min. Cells were stained with the ORO working solution (40% of ORO staining and 60% of milliQ water) for 20 min at 25 °C and examined by an optical microscope (PrimoVert, Zeiss, Jena Germany) to evaluate lipid accumulation. Lipids were extracted from the cells using DMSO and quantified with a microplate reader at 510 nm. Data were expressed as percentage of triglyceride accumulation *vs.* CTRL.

#### 3.2.3. Lipolysis Assay

Lipolytic activity was detected with AdipoLyzeTM Lipolysis Detection Kit (Lonza Inc., Walkersville, MD, USA) according to provider’s instructions. Using a fluorescent kit designed to quantify the glycerol released by cells undergoing lipolysis, the glycerol detection was performed on P20 and A7 cells. An amount of 50 μL of the culture medium supplemented with RR or RS extract was removed from each well and added to a new 96 well plate. Enzyme/detection solution (50 μL) was added in each well followed by further incubation for 1 h in dark. Finally, fluorescence was measured at 570 nm excitation and 595 nm emission wavelength by a fluorescent microplate reader. Orbital shaking for 5 s was applied before measurement. Data were compared with a standard curve of fluorescence obtained from measurements of standard glycerol from 0.0 μM to 108.6 μM.

#### 3.2.4. Apoptosis Assay

Detection of apoptotic cells was done using ApoStrand^TM^ ELISA apoptosis detection kit (Enzo Life Sciences Inc., Farmingdale, NY, USA) was used according to the provider’s instructions. This assay is based on the denaturation of DNA in apoptotic cells by formamide, which reproduces changes in chromatin related to apoptosis. Further the denatured DNA is revealed with a mixture of primary antibody and peroxidase-labeled secondary antibody. P10, P20 and A7 RR and RS-treated cells (30 µg/mL) were fixed for 30 min and dried in an oven at 56 °C for 20 min. Subsequently cells were incubated with formamide at 56 °C for 30 min. Then, blocking solution was added and cells were incubated with antibody mixture for 30 min. After washing with 1X washing buffer, cells were incubated with 100 µL of peroxidase substrate and absorbance was measured using an ELISA plate reader at 405 nm. The apoptotic positive control (single stranded DNA in PBS) was also included in the analysis. Data were expressed as percentage of cell apoptosis *vs* positive CTRL.

#### 3.2.5. TUNEL Assay

To evaluate apoptosis also TUNEL assay was set up on P20 RS and RR-treated cells and P20 CTRL cells grown on glass coverslips. Briefly, 10,000 cells were cultured O/N on sterile coverslip, previously coated with 0.1% gelatin. Then cells were fixed with 2% buffered formalin for 15 min at RT. After incubation with 0.2% Triton X-100 in PBS-Tween (PBST) for 15 min, coverslips were rinsed for 2 min in two changes of PBST. Then cells were blocked for endogenous peroxidase in 3% H_2_O_2_ in PBS for 10 min and after two washes in PBST, the TdT Reaction Buffer containing 1 mM cobalt chloride in 0.2 M sodium cacodylate, 25 mM Tris HCl and 0.25 mg/mL bovine serum albumin pH 6.6, was used in the pre-incubation at RT. Further the specimens were exposed to the TUNEL labeling mix, containing 400 U/µL calf thymus terminal deoxynucleotidyl transferase (TdT) (Roche Diagnostic, Monza, Italy) and 0.5 nmol Biotin-16-dUTP (Roche Diagnostic), in TdT reaction buffer followed by 1 h incubation in a moist chamber at 37 °C. The reaction was stopped with 300 mM NaCl, 30 mM sodium citrate; the specimens were incubated with streptavidin-HRP (Sigma) in PBS for 20 min at RT. For light microscopy detection was performed with 3,3ʹ-diaminobenzidine tetrahydrochloride (DAB solution, Vector Laboratories, Burlingame, CA, USA) as chromogen; the specimens were counterstained with Gill’s hematoxylin, washed in running tap water, dehydrated by passing through graded ethanol cleared in xylene and, finally, mounted with Diamount medium (Diapath, Martinengo, Italy). Cells distinctly stained with a clear positivity to TUNEL were observed under microscope (Leica DM750, Leica Microsystems, Milan, Italy) equipped with a Leica ICC50 HD camera.

#### 3.2.6. RNA Extraction and Adipogenesis PCR Array

Before total RNA extraction treated and CTRL P20 cells were washed with cold 1X PBS. Total RNA extraction was performed with RNeasy kit with QIAzol Lysis Reagent (Qiagen, Milan, Italy), according to the manufacturer’s instructions. To perform PCR array, the cDNA was synthesised from the purified RNA samples according to RT^2^ First Strand kit (Qiagen). Briefly, 10 μL of genomic DNA elimination mixture including 1 μg of the purified RNA was incubated at 42 °C for 5 min. Then the mixture was immediately placed on ice for one minute and added with 20 μL of RT reaction mixture. Tubes were incubated at 42 °C for 15 min and at 95 °C for 5 min. Afterwards, 91 µL of RNase free H_2_O were added to each 30 µL of cDNA synthesis reaction.

The expression profile of adipogenesis was performed using ready to use human Adipogenesis RT^2^ Profiler PCR Array (PAHS-049Z; Qiagen) containing primers for 84 tested, five housekeeping genes and controls for RT and PCR reactions. The synthesized cDNA was used for preparation of reaction mixture according to the instructions of RT² SYBR^®^ Green qPCR Mastermix kit (Qiagen,). Reaction mixture (20 µL), based on CFX96 Real-Time PCR Detection System (Bio-Rad, Milan, Italy), was added to 96 well-plate followed by thermal cycle recommended by manufacturer for Bio-Rad CFX96 (10 min initial denaturation at 95 °C followed by 40 cycles: 15 s at 95 °C, 30 s amplification at 55 °C and 30 s extension at 72 °C). Calculations of contamination with human genomic DNA accordingly to manufacturer instructions showed lack of contamination on all plates. Beta-2-microglobulin (B2M), glyceraldehyde-3-phosphate dehydrogenase (GAPDH), hypoxanthine phosphoribosyltransferase 1 (HPRT1) and ribosomal protein L13a (RPL13A) were chosen from the group of five housekeeping genes as the best and least varying reference genes. β-Actin (ACTB) was not used, since the coefficient of variation of the Ct values was more than two fold than that of other housekeeping genes. The expression of target genes was normalized and ΔCts were calculated by the difference between Ct of target genes and the geometric mean of the four housekeeping genes. Differences between RR and RS samples and CTRL were calculated using the 2^−ΔΔCt^ method [82,83], where 2^−ΔΔCt^ represents the difference of a given target gene in treated cells *vs* CTRL. The *n*-fold expression of a given target gene was calculated as log_2_(2^−ΔΔCt^).

### 3.3. Statistical Analysis

Data were analysed with ANOVA [84]. For cell viability analysis, the model included the amount of RR or RS as fixed effect (4 levels). For apoptosis, triglyceride accumulation and lipolysis assays all models included the fixed effect of treatment (RR, RS and CTRL, 3 levels) and fixed effect of times of incubation that were for apoptosis assay 3 levels (P10, P20 and A7) for triglyceride and lipolysis assays 2 levels (P10 and P20) and their interactions. PCR array data, expressed as log_2_(*n*-fold), were analysed using one sample T test [84]. For a graphical appraisal and for quick identification of changes in PCR array log_2_(*n*-fold), Volcano plot was used and the statistical significance was reported as −Log(*p*-value) at *p* < 0.05.

## 4. Conclusions

In conclusion, these data support the lipolytic and anti-adipogenetic activity of two different commercial extracts of *Rhodiola rosea* in primary human visceral pre-adipocytes during differentiation. In particular, the extract containing salidroside and rosavines (RR) was more effective in regulating the molecular and cellular events of adipogenesis, while RS extract, being richer in salidroside, induced lipolysis and the loss of differentiating cells by apoptosis.

## Supplementary Materials

Supplementary materials can be accessed at: http://www.mdpi.com/1420-3049/20/05/8409/s1.

## Abbreviations

ACACB: acetyl-CoA carboxylase beta
ADIG: adipogenin
ADIPOQ: adiponectin
ADRB2: adrenoceptor beta 2
AGT: angiotensinogen
ANGPT2: angiopoietin 2
AXIN1: axin 1
BMP2: bone morphogenetic protein 2
BMP4: bone morphogenetic protein 4
BMP7: bone morphogenetic protein 7
CCND1: cyclin D1
CDK4: cyclin-dependent kinase 4
CDKN1A: cyclin-dependent kinase inhibitor 1A (p21, Cip1)
CDKN1B: cyclin-dependent kinase inhibitor 1B (p27, Kip1)
CEBPA: CCAAT/enhancer binding protein (C/EBP) alpha
CEBPB: CCAAT/enhancer binding protein (C/EBP) beta
CEBPD: CCAAT/enhancer binding protein (C/EBP) delta
CFD: complement factor D (adipsin)
CREB1: cAMP responsive element binding protein 1
DDIT3: DNA-damage-inducible transcript 3
DIO2: deiodinase, iodothyronine, type II
DKK1: dickkopf WNT signaling pathway inhibitor 1
DLK1: delta-like 1 homolog (*Drosophila*)
E2F1: E2F transcription factor 1
EGR2: early growth response 2
FABP4: fatty acid binding protein 4, adipocyte
FASN: fatty acid synthase
FGF1: fibroblast growth factor 1 (acidic)
FGF10: fibroblast growth factor 10
FGF2: fibroblast growth factor 2 (basic)
FOXC2: forkhead box C2 (MFH-1, mesenchyme forkhead 1)
FOXO1: forkhead box O1
GATA2: GATA binding protein 2
GATA3: GATA binding protein 3
HES1: hes family bHLH transcription factor 1
INSR: insulin receptor
IRS1: insulin receptor substrate 1
IRS2: insulin receptor substrate 2
JUN: jun proto-oncogene
KLF15: Kruppel-like factor 15
KLF2: Kruppel-like factor 2
KLF3: Kruppel-like factor 3
KLF4: Kruppel-like factor 4
LEP: leptin
LIPE: lipase, hormone-sensitive
LMNA: lamin A/C
LPL: lipoprotein lipase
LRP5: low density lipoprotein receptor-related protein 5
MAPK14: mitogen-activated protein kinase 14
NCOA2: nuclear receptor coactivator 2
NCOR2: nuclear receptor corepressor 2
NR0B2: nuclear receptor subfamily 0, group B, member 2
NR1H3: nuclear receptor subfamily 1, group H, member 3
NRF1: nuclear respiratory factor 1
PPARA: peroxisome proliferator-activated receptor alpha
PPARD: peroxisome proliferator-activated receptor delta
PPARG: peroxisome proliferator-activated receptor gamma
PPARGC1A: peroxisome proliferator-activated receptor gamma, coactivator 1 alpha
PPARGC1B: peroxisome proliferator-activated receptor gamma, coactivator 1 beta
PRDM16: PR domain containing 16
RB1: retinoblastoma 1
RETN: resistin
RUNX1T1: runt-related transcription factor 1
RXRA: retinoid X receptor, alpha
SFRP1: secreted frizzled-related protein 1
SFRP5: secreted frizzled-related protein 5
SHH: sonic hedgehog
SIRT1: sirtuin 1
SIRT2: sirtuin 2
SIRT3: sirtuin 3
SLC2A4: solute carrier family 2 (facilitated glucose transporter), member 4
SRC: v-src avian sarcoma (Schmidt-Ruppin A-2) viral oncogene homolog
SREBF1: sterol regulatory element binding transcription factor 1
TAZ: tafazzin
TCF7L2: transcription factor 7-like 2 (T-cell specific, HMG-box)
TSC22D3: TSC22 domain family, member 3
TWIST1: twist family bHLH transcription factor 1
UCP1: uncoupling protein 1 (mitochondrial proton carrier)
VDR: vitamin D (1,25- dihydroxyvitamin D3) receptor
WNT1: wingless-type MMTV integration site family, member 1
WNT10B: wingless-type MMTV integration site family, member 10B
WNT3A: wingless-type MMTV integration site family, member 3A
WNT5A: wingless-type MMTV integration site family, member 5A
WNT5B: wingless-type MMTV integration site family, member 5B
