# Supplementary material for: Effects of Two Different Rhodiola rosea Extracts on Primary Human Visceral Adipocytes
Source: Molecules. 2015 May 11;20(5):8409–28. doi: 10.3390/molecules20058409 (PMC6272273; doi:10.3390/molecules20058409)
Supplement: Supplementary file 1 [file molecules-20-08409-s001.pdf]

Supplementary Materials

Table S1. Adipogenesis PCR array results. Mean of $\log_2(n\text{-fold}) \pm$ standard error of the mean (SEM) and *p*-value. Total RNA from three independent experiments, one per each donor, was isolated for both CTRL cells and cells treated with RR or RS extracts.

**Gene**	**log₂(*n*-fold)**			
	**RS extract**		**RR extract**	
	**mean (SEM)**	***p*-value**	**mean (SEM)**	***p*-value**
ACACB	−1.81 (1.042)	0.648	0.16 (0.462)	0.760
ADIG	3.72 (0.940)	1.234	1.66 (0.109)	0.004
ADIPOQ	1.91 (0.646)	1.011	0.21 (0.105)	0.188
ADRB2	−2.84 (0.712)	1.241	1.38 (0.128)	0.008
AGT	−0.43 (0.268)	0.606	−0.31 (0.111)	0.108
ANGPT2	0.32 (1.060)	0.100	−1.15 (0.078)	0.004
AXIN1	0.98 (1.045)	0.351	0.19 (0.134)	0.290
BMP2	3.03 (0.830)	1.170	0.44 (0.216)	0.179
BMP4	1.35 (0.357)	1.200	0.77 (0.522)	0.278
BMP7	1.71 (0.353)	1.396	2.03 (0.039)	0.000
CCND1	−2.96 (0.979)	1.027	−0.95 (0.028)	0.001
CDK4	−0.75 (0.985)	0.279	−0.06 (0.129)	0.689
CDKN1A	−0.27 (0.995)	0.092	0.27 (0.088)	0.090
CDKN1B	−1.45 (0.981)	0.558	−0.77 (0.138)	0.031
CEBPA	−0.86 (0.951)	0.337	0.91 (0.206)	0.047
CEBPB	−0.92 (1.025)	0.333	−0.79 (0.444)	0.218
CEBPD	−0.12 (1.002)	0.038	−0.90 (0.130)	0.020
CFD	−1.34 (0.978)	0.516	0.24 (0.194)	0.344
CREB1	−0.08 (0.629)	0.043	−0.94 (0.064)	0.005
DDIT3	−2.97 (0.980)	1.027	−1.22 (0.178)	0.020
DIO2	−1.41 (0.648)	0.793	−2.47 (0.361)	0.021
DKK1	−1.74 (0.997)	0.653	2.85 (0.161)	0.003
DLK1	1.17 (1.010)	0.437	1.92 (0.284)	0.021
E2F1	−0.70 (0.985)	0.258	1.04 (0.131)	0.015
EGR2	3.30 (0.711)	1.362	2.07 (0.126)	0.004
FABP4	−2.88 (0.647)	1.328	−3.19 (0.039)	0.000
FASN	−1.26 (0.984)	0.483	0.07 (0.356)	0.865
FGF1	−1.56 (0.978)	0.600	−0.55 (0.263)	0.173
FGF10	4.33 (0.820)	1.468	2.22 (0.138)	0.004
FGF2	−1.61 (0.293)	1.502	−1.30 (0.146)	0.012
FOXC2	0.01 (0.986)	0.003	0.57 (0.362)	0.258
FOXO1	−2.37 (0.980)	0.866	−0.13 (0.444)	0.795
GATA2	2.54 (0.533)	1.384	0.98 (0.122)	0.015
GATA3	2.54 (0.983)	0.911	0.92 (0.184)	0.038
HES1	−0.13 (0.621)	0.068	−0.26 (0.118)	0.155
INSR	−1.41 (0.978)	0.543	−0.50 (0.234)	0.166
IRS1	−1.49 (0.978)	0.575	0.09 (0.151)	0.629
IRS2	1.61 (1.029)	0.588	0.34 (0.376)	0.463
JUN	−1.13 (0.978)	0.435	−0.01 (0.334)	0.976
KLF15	5.65 (1.038)	1.492	2.24 (0.026)	0.000

Table S1. *Cont.*

Gene	log₂(*n*-fold)			
	RS extract		RR extract	
	mean (SEM)	*p*-value	mean (SEM)	*p*-value
KLF2	0.80 (0.546)	0.553	1.00 (0.208)	0.040
KLF3	−1.97 (0.978)	0.741	−0.28 (0.434)	0.583
KLF4	−2.24 (0.978)	0.828	−0.01 (0.384)	0.988
LEP	−1.28 (0.986)	0.492	−0.71 (0.258)	0.111
LIPE	2.68 (0.671)	1.242	0.23 (0.071)	0.086
LMNA	0.20 (1.013)	0.063	0.12 (0.352)	0.772
LPL	1.44 (0.624)	0.831	1.01 (0.108)	0.011
LRP5	−0.58 (1.022)	0.202	1.00 (0.391)	0.125
MAPK14	−1.44 (0.979)	0.554	−0.83 (0.436)	0.198
NCOA2	−0.73 (0.982)	0.271	−0.42 (0.201)	0.172
NCOR2	−0.11 (1.006)	0.035	−0.16 (0.319)	0.666
NR0B2	2.81 (0.373)	1.766	1.54 (0.102)	0.004
NR1H3	−1.00 (0.978)	0.385	0.01 (0.208)	0.959
NRF1	1.38 (0.627)	0.798	0.86 (0.208)	0.054
PPARA	−1.99 (0.996)	0.737	−0.44 (0.079)	0.031
PPARD	−0.81 (0.996)	0.301	−0.10 (0.048)	0.175
PPARG	−2.52 (1.060)	0.851	−0.98 (0.111)	0.013
PPARGC1A	−0.75 (0.981)	0.282	−1.09 (0.341)	0.085
PPARGC1B	−1.53 (0.772)	0.730	0.22 (0.106)	0.169
PRDM16	2.55 (0.978)	0.917	2.31 (0.068)	0.001
RB1	−3.20 (1.002)	1.068	−2.16 (0.346)	0.025
RETN	3.08 (0.737)	1.277	1.78 (0.169)	0.009
RUNX1T1	−0.83 (0.627)	0.502	−1.25 (0.094)	0.006
RXRA	−1.12 (0.978)	0.433	−0.24 (0.122)	0.191
SFRP1	1.23 (1.017)	0.455	−1.43 (0.184)	0.016
SFRP5	4.17 (0.938)	1.328	2.71 (0.101)	0.001
SHH	3.96 (1.076)	1.176	1.45 (0.174)	0.014
SIRT1	0.66 (0.974)	0.247	−0.43 (0.071)	0.026
SIRT2	0.33 (0.077)	1.291	0.40 (0.271)	0.282
SIRT3	−0.61 (0.979)	0.224	−1.09 (0.244)	0.047
SLC2A4	0.03 (0.627)	0.013	−0.71 (0.138)	0.035
SRC	−0.42 (0.980)	0.149	0.47 (0.224)	0.172
SREBF1	−0.70 (0.979)	0.260	−0.39 (0.161)	0.136
TAZ	1.22 (1.024)	0.449	0.99 (0.041)	0.002
TCF7L2	−1.96 (0.988)	0.732	0.00 (0.208)	0.993
TSC22D3	0.08 (0.993)	0.024	−0.11 (0.048)	0.140
TWIST1	−2.59 (0.996)	0.917	−0.68 (0.201)	0.077
UCP1	1.64 (1.484)	0.416	−1.54 (0.358)	0.050
VDR	−1.27 (1.019)	0.470	−0.34 (0.154)	0.157
WNT1	3.73 (0.686)	1.493	0.00 (0.000)	0.000
WNT10B	0.43 (0.981)	0.151	2.87 (0.208)	0.004
WNT3A	3.19 (1.022)	1.049	1.15 (0.174)	0.031
WNT5A	−1.13 (0.978)	0.437	−1.07 (0.124)	0.013
WNT5B	5.85 (0.973)	1.576	2.87 (0.224)	0.006
